# Novel magnetic iron–nickel/poly(ethersulfone) mixed matrix membranes for oxygen separation potential without applying an external magnetic field

**DOI:** 10.1038/s41598-022-16979-6

**Published:** 2022-08-11

**Authors:** Norhan Nady, Noha Salem, Sherif H. Kandil

**Affiliations:** 1 Polymeric Materials Research Department, City of Scientific Research and Technological Applications (SRTA-City), Borg El-Arab City, Alexandria, 21934 Egypt; 2 Department of Materials Science, Institute of Graduate Studies and Research, Alexandria University, Alexandria, 21526 Egypt

**Keywords:** Materials science, Nanoscience and technology

## Abstract

This work presents novel magnetic mixed matrix poly(ethersulfone) (PES) membranes that combine the advantages of low-cost common PES polymer and low-cost iron–nickel magnetic alloys. Moreover, the presented magnetic mixed matrix PES membranes were fabricated and used without applying an external magnetic field during either the membrane casting or the separating process. The fabricated magnetic membranes were prepared using the phase inversion technique and *N*-methylpyrrolidone and *N,N*‐Dimethylformamide solvents mixture with volumetric ratio 1:9 and Lithium chloride as an additive. The used iron–nickel magnetic alloys were prepared by a simple chemical reduction method with unique morphologies (Fe_10_Ni_90_; starfish-like and Fe_20_Ni_80_; necklace-like). The fabricated membranes were characterized using Scanning Electron Microscope (SEM) and Scanning-Transmission Electron Microscope (STEM) imaging, energy dispersive X-ray (EDX), Thermogravimetric (TGA), and X-ray diffraction (XRD). Also, static water contact angle, membrane thickness, surface roughness, membrane porosity, membrane tensile strength as well as Vibrating Sample Magnetometer (VSM) analysis and oxygen transition rate (OTR) were determined. Moreover, the effect of alloy concentration and using Lithium chloride as an additive on the properties of the fabricated blank PES and magnetic mixed matrix PES membranes were studied. The presented novel magnetic mixed matrix PES membranes have high coercivity up to 106 (emu/g) with 3.61 × 10^–5^ cm^3^/cm^2^·s OTR compared to non-oxygen permeable blank PES membranes. The presented novel magnetic mixed matrix PES membranes have good potential in (oxygen) gas separation.

## Introduction

The separation of air into its components generally is carried out for industrial and medical use. The separation of binary gas mixtures is especially in wide demand to produce valuable gases for numerous applications and mitigate pollution. Hydrogen, oxygen, and nitrogen gases are considered the most valuable gases of particular importance as the obtained individual pure gases can be contributed efficiently in several areas^1^. Oxygen-enriched air has various medical, chemical, and industrial applications, for example, it is used for combustion enhancement in oxy-fuel combustion by increasing the burning velocities^2^, catalysts regeneration in fluid catalytic cracking^3^, indoor air quality improvement^4,5^, sewerage treatment plants^6,7^, and medical treatments^8,9^. Meanwhile, the nitrogen-enriched air can be applied in food storage^10,11^, fires control^12,13^, oil recovery^14,15^, and water drainage^16^.

Conventional techniques that have been used for O_2_/N_2_ gas separation are cryogenic distillation^17,18^ and pressure swing adsorption (PSA)^19–21^. Both techniques are commercial technology where oxygen and nitrogen can be produced in a substantially adequate amount and high purity, however, they are limited by their complexity, large space requirements, high cost as well as high energy consumption^22^. Membrane-based gas separation has gained the special attention of researchers in the past decades. It offers numerous advantages over the conventional methods in terms of energy consumption, footprint, small space, environmental friendliness, relatively capital and operating cost, and ease of operation^23–25^.

Membranes for gas separations are classified into: organic (polymeric), inorganic, mixed matrix (composite) membranes (MMMs), and other recently developed membranes such as ionic liquid supported membranes (ILSM)^26^, polymers with intrinsic microporosity (PIMs)^27^, metal–organic framework (MOF)^28^, and thermally rearranged (TR) polymers^29^. The drawbacks of polymeric membranes include an inherent tradeoff between permeability and selectivity, as well as lower thermal and chemical stability compared to inorganic membranes. Inorganic membranes have higher separation efficiency than polymeric membranes and can withstand high-temperature separation processes; however, their separation is inversely proportional to the pressure of the feed gas as well as poisoning possibilities^30^. To improve the applications of membranes in gas separation, novel materials/mixed matrix membranes (MMMs) can combine the advantages of both the polymeric matrix and the inorganic filler, and minimize the drawbacks of both components to be the key for improving membrane-based gas separation.

Recently, the integration of magnetic nanoparticles with polymers is a new class of MMMs that has been addressed widely in N_2_/O_2_ gas separation. The separation is based on the difference between oxygen and nitrogen properties in a magnetic field; oxygen is paramagnetic whereas nitrogen is diamagnetic. The separation is carried out by the effect of a gradient magnetic field on oxygen molecules^31,32^. Magnetic neodymium powder was dispersed in an ethyl cellulose membrane and was used for N_2_/O_2_ separation in the presence of an external magnetic field, which resulted in 56% oxygen enrichment in permeate for the magnetic induction of 2.25 mT. Moreover, the grading in the magnetic field that resulted in 65% enrichment of oxygen in air depends on the magnetic field direction and increased with the increase in magnetic induction^32^. In another research, poly(ethersulfone) (PES) membrane surface was coated with polydimethylsiloxane and then coated with FluidMAG-PAD^31^; PES was used as a support layer for the main separating layers of PDMS and commercial magnetic iron oxides nanoparticles impeded in polyacrylamide polymer and the produced membrane that consists of Polyacrylamide/PDMS/PES and PDMS/PES/Polyacrylamide showed high oxygen selectivity. Also, iron oxides (Fe_3_O_4_) nanoparticles was applied in both ethyl cellulose and poly(2,6-dimethyl-1,4-phenyleneoxide) (PPO)^33^ polymers. Magnetic nanoparticle loaded amount, size, distribution, and agglomeration effects on the membrane performance in oxygen separation were studied^34^. Recently, Fe pillared Cloisite 15A (P–C15A) was dispersed in a polysulfone (PSf) matrix with different loading percentages^35^. Magnetic double-layer MMMs consisting of PES/Pebax-1657-BaFe_12_O_19_ nanoparticles with 18 and 24 wt% filler fabricated by co-casting methods showed enhanced selectivity of O_2_/N_2_ gases 4 and 4.01; respectively, in the presence of H = 0.5 T at 25 °C^36^. Also, PSf embedded with 10 wt% carbonyl iron powders (CIPs) improved O_2_ permeability and selectivity by 436% and 41%; respectively, compared to the pure PSf membrane in the presence of a 570 mT magnetic field during permeation tests^37^.

Nanocrystalline iron-based alloy is among the most attractive ferromagnetic metallic nanomaterials for electromagnetic applications. The attention to this alloy is attributed to its unique magnetic properties, excellent mechanical stiffness, strength and thermal dimensional stability (rigidity), good electrical properties, and reasonable cost^38,39^. Although researchers have succeeded in preparing many magnetic MMMs from different polymers and inorganic magnetic nanofiller materials, there are several defects that should be addressed including (1) very expensive magnetic filler (praseodymium or neodymium) that impedance membrane application on a large scale, (2) the used iron-oxide nanoparticles as filler in magnetic MMMs needs applying an external magnetic field during the separation process because the iron-oxide nanoparticles lose their magnetization once the magnetic field moved away, (3) lack of homogenous dispersion of the nanofillers inside the formed polymeric matrix.

From this motivation, this work combines the advantages of both low-cost common polymers and low-cost simple-prepared inorganic alloys/fillers and enables the use of novel magnetic mixed matrix membranes on a wider range and more efficient in (Oxygen) gas separation. The used magnetic alloys were prepared by a simple chemical reduction method with different and unique morphologies (Fe_10_Ni_90_; starfish-like and Fe_20_ Ni_80_; necklace-like) that are characterized by their high magnetic properties, high purity (99.9%), and easy preparation on large scale. Moreover, the used magnetic alloys are ferromagnetic and have high coercivity (i.e., the intensity of the applied magnetic field required to demagnetize the material; high coercivity is a sign of permanent magnetization) and can be used without applying a magnetic field during separation; that resulted in an efficiency to be applied on a large industrial scale. The novel membranes were prepared by using the casting method which is simple, inexpensive, and easy to control.

The prepared blank PES and the mixed matrix PES membranes were characterized using Scanning Electron Microscope (SEM) and Scanning-Transmission Electron Microscope (STEM) imaging, energy dispersive X-ray (EDX), Thermogravimetric (TGA), and X-ray diffraction (XRD). Also, static water contact angle, membrane thickness, surface roughness, membrane porosity using ethanol and water, membrane tensile strength, Vibrating Sample Magnetometer (VSM) analysis as well as Oxygen Transition Rate (OTR) were determined. Moreover, the effect of alloy concentration and using Lithium chloride as an additive on the properties of the prepared blank PES and magnetic mixed matrix PES membranes were studied.

## Materials and methods

### Materials

**Figure 1:**
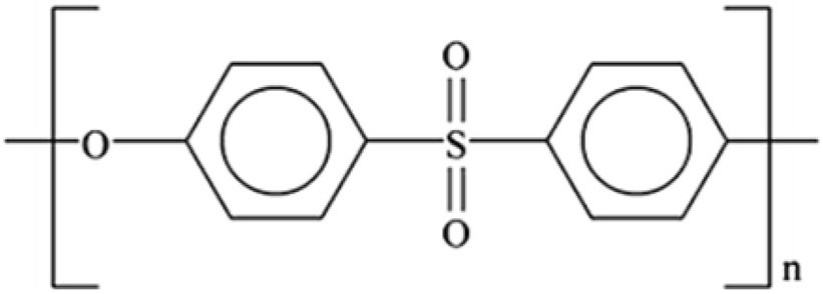
The structure of poly(ethersulfone) (PES) polymer.

Poly(ethersulfone) (PES) Ultrason E 6020P (glass transition temperature T_g_ = 225 °C and a molecular weight (Mw) of 58,000 g/mol, polymer density = 1.37 g/cm^3^) was obtained from BASF chemical company (Ludwigshafen, Germany). *N,N*‐Dimethylformamide (DMF) (HPLC grade, 99.8%), *N*-methylpyrrolidone (NMP) (anhydrous, > 99% purity), and Lithium chloride (anhydrous, > 99% purity) were purchased from Sigma-Aldrich. Nickel chloride hexahydrate (NiCl_2_·6H_2_O, 98%), and Ferrous chloride tetrahydrate (FeCl_2_·4H_2_O, 99.99%) were purchased from Alfa Aesar (Thermo Fisher Scientific, United States). Hydrazine hydrate reducing agent (N_2_H_4_·H_2_O, 99%) was obtained from Fisher (Horsham, UK). Sodium hydroxide (NaOH, 98%) catalyst was purchased from Trading Dynamic co. TDC (Cairo, Egypt). Distilled water was used as a solvent for nanostructured alloys syntheses. Figure 1 illustrates the chemical structure of PES polymer.

### Methods

#### Preparation of magnetic nanostructured iron–nickel alloys using the chemical reduction method

**Figure 2:**
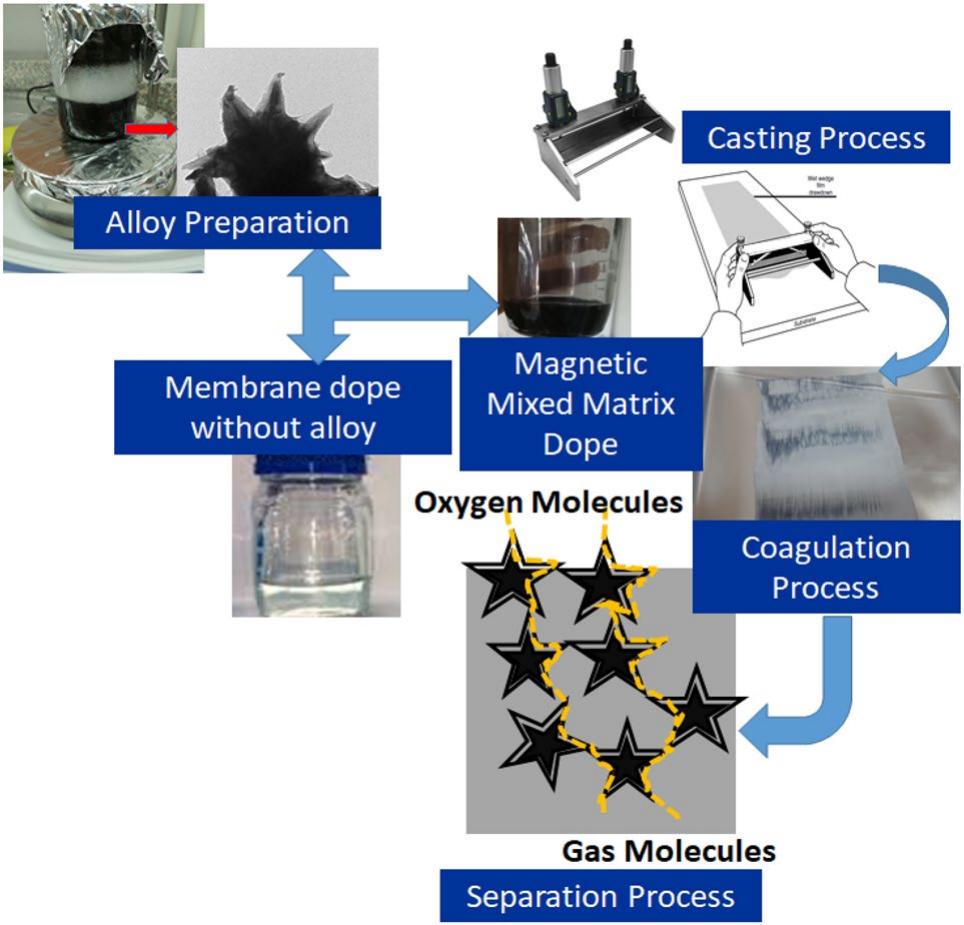
A schematic representation of the preparation procedure of both the fillers; for example, Fe_10_Ni_90_ alloy, and the mixed matrix poly(ethersulfone) (PES) membranes.

The iron–nickel alloys were prepared as described in our previous paper^40^. Briefly; ferrous chloride and nickel chloride were dissolved to prepare 80 ml of 0.1 M aqueous solution containing both iron and nickel ions in different weight ratios. Hot reduction solution of hydrazine (99%) that was prepared in an alkaline solution of sodium hydroxide in proportions of 1:5, was added to the metal solution with strong stirring up to 1500 rpm and a temperature of 95–98 °C as shown in Fig. 2. The chemical reduction reaction time is about 15 min. A black precipitate was separated by a magnet and was washed well with distilled water until the neutral medium was reached and was dried in a vacuum oven for a period of a day at a temperature of 35 °C. Two molar ratios were prepared; Fe_10_Ni_90_ and Fe_20_Ni_80_. Figure 2 shows a schematic representation of the preparation procedure of the fillers and the mixed matrix PES membranes as well as the (oxygen) separation process as described in the next sections.

### Preparation of magnetic mixed matrix PES membranes

Poly(ethersulfone) (PES) was prepared in a 20 ml polymer solution by mixing 18 wt.% PES, 1 wt.%% Lithium chloride in solvents mix of 90 vol.% dimethylformamide (DMF) and 10 vol.% N-methyl pyrrolidone (NMP), with mixing until fully dissolving and formation of a viscous homogeneous solution. In another separate bottle, the prepared magnetic alloys (Fe_10_Ni_90_; starfish-like and Fe_20_Ni_80_; necklace-like) were first dispersed in 5 ml of 10% NMP and 90% DMF solvents mix using ultrasonication for 30 min at room temperature, and then they were added to PES polymer solutions after magnet removal using ultrasonication of the formed mixed matrix dopes two times for 15 min at room temperature. The air bubbles were removed from the mixed matrix dopes by degassing for 20 min, then the mixed matrix PES membranes were prepared by casting method using a doctor casting knife adjusted at 350 µm. Then, the as-cast magnetic mixed matrix PES membranes were immersed in 2.5 l of distilled water with ethanol/methanol at room temperature for 2 h as shown in Fig. 2. Finally, the prepared magnetic mixed matrix PES membranes were dried at room temperature. Magnetic mixed matrix PES dope was prepared with both low (0.05%) and high (2%) concentrations of iron–nickel magnetic fillers. Table 1 shows the coded name and composition of the fabricated blank PES and magnetic mixed matrix PES membranes.

**Table 1:** Coded name and composition of the fabricated blank and magnetic mixed matrix poly(ethersulfone) (PES) membranes.

No.	Membrane code	PES concn. (wt%)	Filler concn. (wt%)	Filler shape	LiCl concn. (wt%)
1	PES blank WO	18	0	No filler	0.0
2	PES blank W	18	0	No filler	0.1
3	PES 1090 0.05% WO	18	0.05	Starfish-like	0.0
4	PES 1090 0.05% W	18	0.05		0.1
5	PES 1090 2.0% WO	18	2.0		0.0
6	PES 1090 2.0% W	18	2.0		0.1
7	PES 2080 2.0% WO	18	2.0	Necklace-like	0.0
8	PES 2080 2.0% W	18	2.0		0.1

### Membrane characterization

#### Membrane porosity

The porosity (ε) of the fabricated magnetic MMMs was obtained by measuring the wet and dry weights of the membrane samples. The wet weight of the membrane sample was measured after immersing it in ethanol or water for 15 min. The dry weight of the sample was measured after drying the sample using an oven at 60 °C for 24 h. The membrane porosity was determined using the following equation^41^:

1 $$\varepsilon = \frac{\frac{{m}_{w}-{m}_{d}}{{\rho }_{e}}}{\left[\frac{{m}_{w}-{m}_{d}}{{\rho }_{e}}\right]+\frac{{m}_{d}}{{\rho }_{P}}},$$

where ${m}_{w}$ is the wet membrane weight (g), ${m}_{d}$ is the dry membrane weight (g), ${\rho }_{e}$ is the density of ethanol or water (g/cm^3^) and ${\rho }_{P}$ is the density of the polymer (g/cm^3^).

### Static water contact angle

The static water contact angle for membranes' samples was measured using Goniometer model 500-F1 coupled with a video camera and image analysis software. The membrane samples were fixed on a glass slide and a water droplet of (7 µl) was dropped on different spots of the membrane surface. The membrane sample was analyzed using the captured images at consecutive time frames and the right and left contact angles were estimated using the image analysis software and the mean value was determined. The reported value was the average of nine readings on three different membrane samples for each composition.

### The oxygen transmission rate (OTR)

The oxygen transmission rate (OTR) was measured using N530-B gas permeability analyzer from GBPI Equipment CO. Ltd., China, according to the standard of ISO 15105-1. ASTM D1434, YBB00082003, JISK7126-A, and GB/T 1038 were used for the evaluation of the oxygen gas transmission rate (OTR) by differential pressure method. The membrane was fixed in the middle of test chamber to separates chamber into upper room and lower room, with keeping a constant pressure difference; the initial pressure for the upper and lower room are 100 Kpa and 10 Pa, respectively. Oxygen gas molecules would penetrate through sample from higher pressure room into lower pressure room. Gas permeability is measured by detecting the pressure change in lower pressure room and calculating the gas transmission rate.

### Membrane thickness

An average of ten measurements at different points on three different membranes was calculated using a micrometer (range 0–25 mm, precision: 2 µm, HDT, China).

### Membrane surface roughness

A surface roughness tester (SJ-201 P, Mitutoyo, Kanagawa, Japan) was used to measure the membrane roughness. The instrument was calibrated by measuring the roughness of the used glass plate to fix the membrane samples on it. The average of nine measurements of three different membrane samples that were prepared from three independent membrane dopes for the same membrane composition was recorded.

### Membrane tensile strength

Both the blank PES and mixed matrix PES membranes were cut in a dumbbell shape. The length of each membrane was 37 mm, the gauge length of the membranes was about 16 mm; the width was 13 mm at the top and 7.2 mm (narrowest) at the middle of the membrane, to force a fracture in the middle of the sample. Tensile testing of the films was performed with the Texture Analyzer T2 (Stable Micro Systems, Ltd., Surrey, United Kingdom), at a constant crosshead speed of 0.1 mm/s. Stress–strain curves were calculated from load–elongation curves measured for five samples from two membranes prepared from two prepared dopes for each membrane composition.

### X-ray diffraction (XRD) analysis

X-ray diffraction (XRD) was employed to characterize the synthesized alloys and the magnetic mixed matrix poly(ethersulfone) membranes. XRD measurements were carried out on a Shimadzu XRD-7000 diffractometer (Kyoto, Japan, 45 kV, 30 mA; CuKα+ Ni-filtered radiation, λ = 0.15406 nm). The 2θ range was 5°–80°, at a scanning rate of 4°/min and a scanning step of 0.026°.

### Thermogravimetric analysis (TGA)

Thermal gravimetric studies of both the blank PES and the magnetic mixed matrix PES membranes were carried out using a thermal gravimetric analyzer (Shimadzu TGA-50, Nishinokyo Kuwabara-Cho, Nakagyo-ku, Kyoto, Japan). The samples were scanned over a temperature range from 25 to 1000 °C at a 10 °C/min temperature gradient under nitrogen flow.

### Scanning electron microscopy (SEM) imaging and energy dispersive X-ray (EDX) analysis

Both the blank PES and magnetic mixed matrix PES membranes were cut using a very sharp shaving blade and were then coated with gold, and imaged at a voltage of 20 kV and a resolution of 1280 × 960 pixels using Scanning electron microscopy (Joel Jsm 6360LA, Akishima, Japan). For cross-section imaging; the membrane samples were immersed in liquid nitrogen and were fractured to be processed for gold coating before imaging. The chemical compositions were determined by an area analysis using energy-dispersive X-ray spectroscopy (EDX) system equipped with SEM.

### Scanning-transmission electron microscope (STEM) imaging and energy dispersive X-ray (EDX) analysis

The fabricated blank PES and magnetic matrix PES membranes were inspected by Transmission electron microscopy (TEM, 2100Plus, JEOL Ltd., Tokyo, Japan), operated at 200 kV. For the nanostructured alloys, they were deposited on a cupper-grid-supported transparent carbon foil. To image the membranes with TEM, membranes samples were frozen inside epoxy blocks (Epon 812) embedding Resin (Mollenhauer, Germany), and a very thin layer was cut by using PowerTom Ultramicrotomes (RMS Boeckeler, Boeckeler Instruments Inc., Tucson, Arizona, USA). The chemical compositions were estimated by an area analysis using energy-dispersive X-ray spectroscopy (EDX) system equipped with STEM.

### Vibrating sample magnetometer (VSM) analysis

A vibrating sample magnetometer (VSM, Lake Shore 7410, USA) was used to measure the room-temperature magnetic properties of the nanostructured iron–nickel alloys and the fabricated blank PES and magnetic mixed matrix PES membranes. The applied field was − 20 ≤ H ≤ 20 kOe. For magnetization measurements, the membranes were tied and fixed in a small cylindrical plastic holder between the magnetic pools.

## Results and discussions

**Figure 3:**
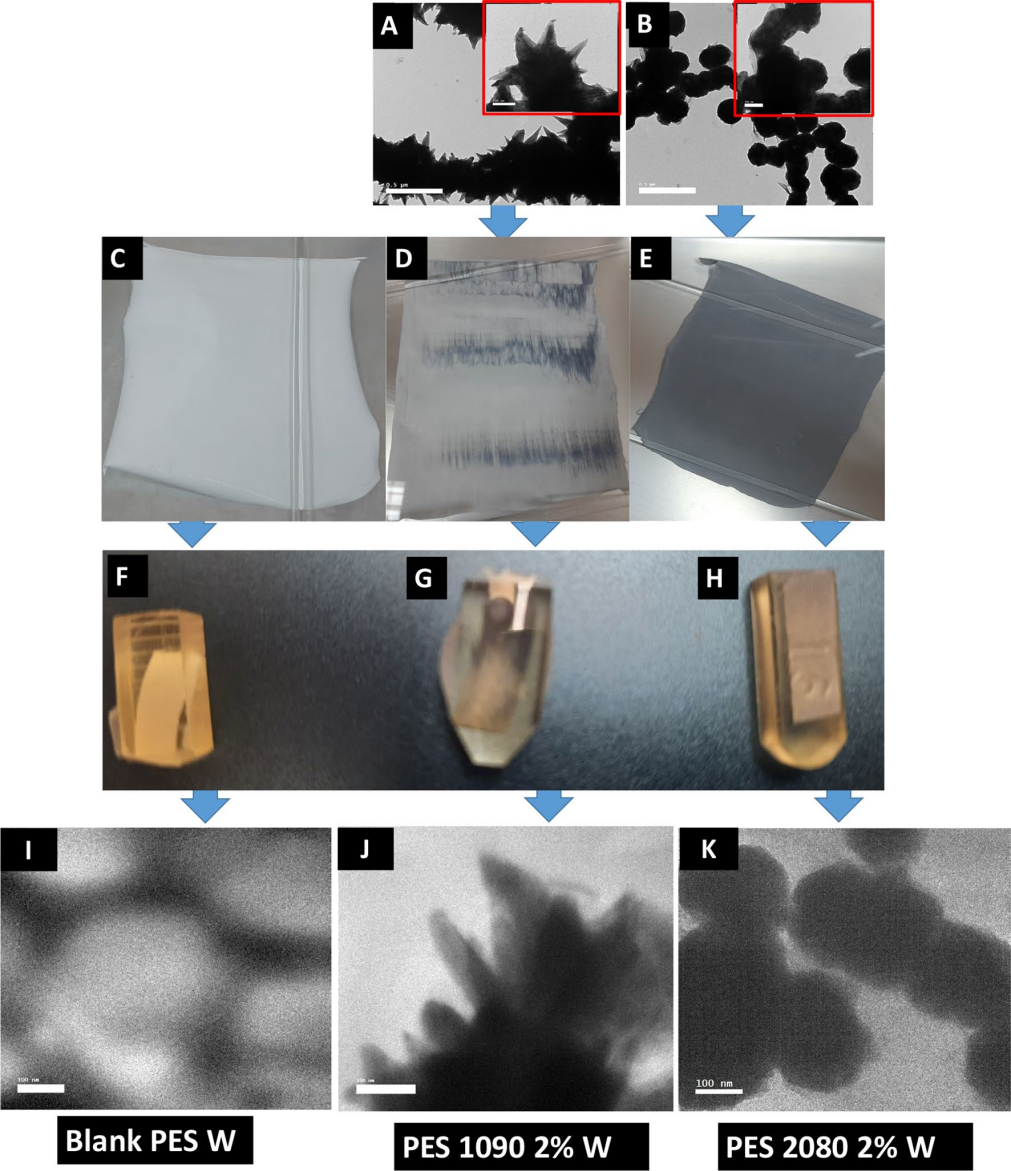
TEM images (0.5 µm and 100 nm bar scales) of the used iron–nickel alloys; starfish-like Fe_10_Ni_90_ (**A**) and necklace-like Fi_20_Ni_80_ (**B**). Photos of the Blank PES (**C**) and the magnetic mixed matrix PES membranes; (**D**) Fe_10_Ni_90_ and (**E**) Fi_20_Ni_80_, and Photos of the prepared epoxy blocks contained the fabricated magnetic mixed matrix PES membranes (blank PES; **F**, mixed matrix PES membranes; **G,H**). TEM images (100 nm bar scale) of the blank PES (**I**) and the mixed matrix PES membranes (**J,K**) inside the epoxy blocks.

In this work, two iron–nickel alloys; starfish-like Fe_10_Ni_90_ and necklace-like Fi_20_Ni_80_, were prepared according to the our previous work^40^ and both of them have a unique microstructure as shown in Fig. 3A,B. These two different distinguished magnetic alloys and their unique magnetic properties were previously presented and discussed^40^. Incorporation of these two magnetic alloys as filler inside the PES blank; Fig. 3C, resulted in the formation of homogeneous magnetic mixed matrix PES membranes as shown in Fig. 3D,E using Fe_10_Ni_90_ and Fi_20_Ni_80_ alloys, respectively. After embedding the membranes inside epoxy blocks (Epon 812; Fig. 3F–H) and they were cut into a very thin layer that was imaged by TEM. The blank PES and the magnetic mixed matrix PES membranes are shown in Fig. 3I–K, respectively, which proved that the blank PES membrane does not contain any fillers and confirm keeping the same morphologies of the used iron–nickel alloys inside the mixed matric PES membranes. What is noticed is the permanent magnetic Fe_10_Ni_90_ alloy was targeted and aligned toward the direction of the casting knife moving. This can be attributed to the magnetic moment of particles and the resulting dipolar interaction that could overcome the thermal motion of particles. In previous works^42^, usually, an external magnetic field was applied; a stable magnetic field with a range of induction 0–40 mT, during the membrane casting, to inhibit the magnetic particle sedimentation and to enhance the proper particle arrangement. Here, in this work. The used casting knife attracted the alloys near/into the surface. This can explain the observation that the contained magnetic fillers were pulled up to the membrane surface in the direction of the knife move. This actually was noticeable to the naked eyes, especially with the Fe_10_Ni_90_ alloy.

### Membranes microstructure

**Figure 4:**
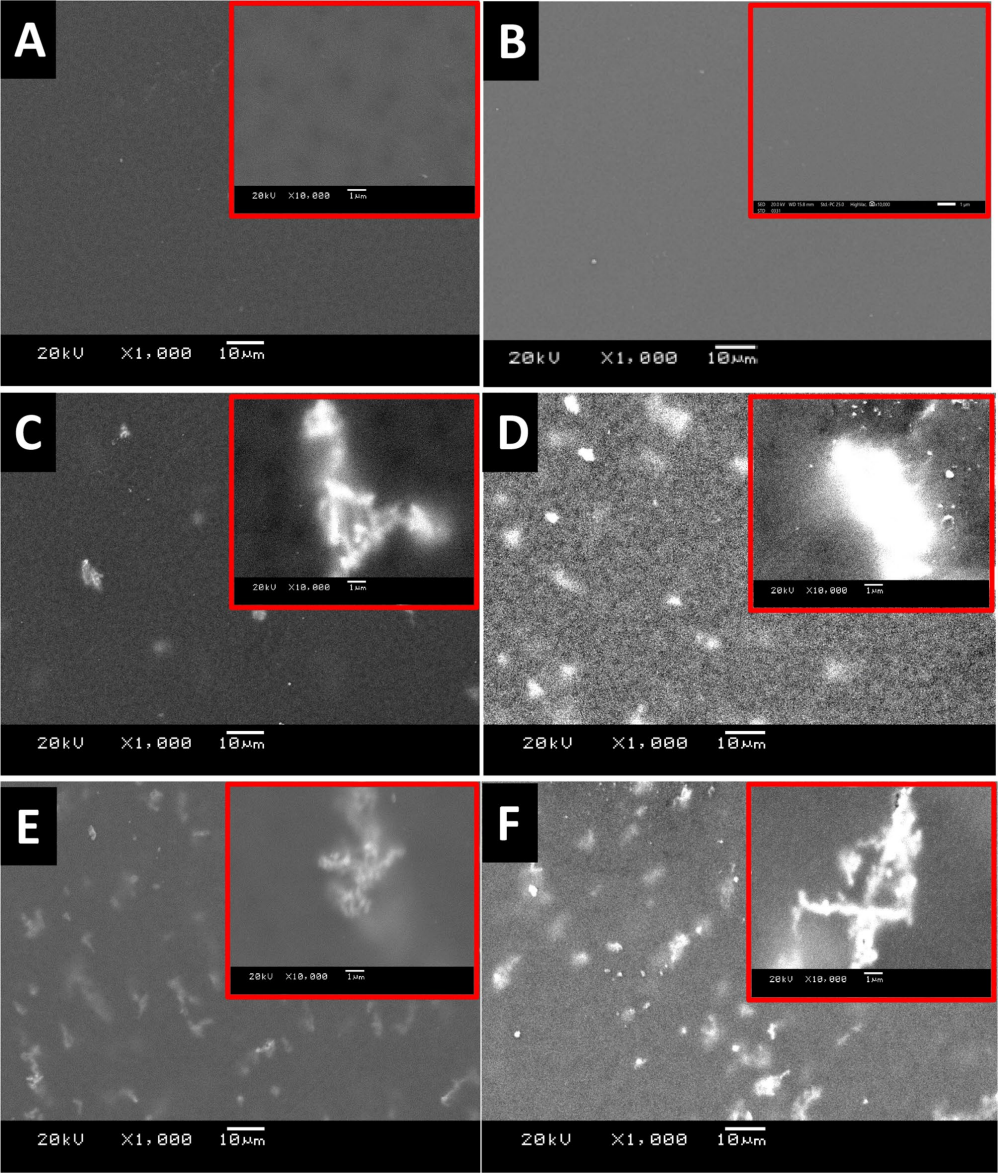
SEM images of the blank PES and mixed matrix PES membranes that were prepared using 1 NMP: 9 DMF solvents mix without (**A,C,E**) and with (**B,D,F**) 0.1 wt% of Lithium chloride. The blank PES membrane (**A,B**), the mixed matrix PES membranes using Fe_10_Ni_90_ (**C,D**), and Fe_20_Ni_80_ (**E,F**) alloys were imaged at × 1000 and × 10,000 magnifications.

The factors contributing to the separation performance of a membrane in terms of permeability and selectivity are the membrane morphology and thickness. In principle, the desired membrane morphology in the gas separation is a spongy structure with considerably low membrane thickness. The electron microscope images of membranes’ surfaces of both the blank PES and the mixed matrix PES membranes were imaged and shown in Fig. 4. The blank PES membranes appear dense top layer without alloys (fillers). The imaging process of the mixed matrix PES membranes was very difficult due to the effect of the high magnetization of the alloys on the microscope. The taken images reveal that the magnetic alloys were dispersed in the PES matrix. The embedded alloys appear very bright, especially with Fe_10_Ni_90_ magnetic alloy (Fig. 4C,D). The non-dissolved traces of the used Lithium chloride may assist the brightness of the used alloys (Fig. 3D; Fe_10_Ni_90_ and 3F; Fe_20_Ni_80_ alloys, respectively). This brightness of the alloys is enhanced with the projection of a tiny part on the membrane surface.

**Figure 5:**
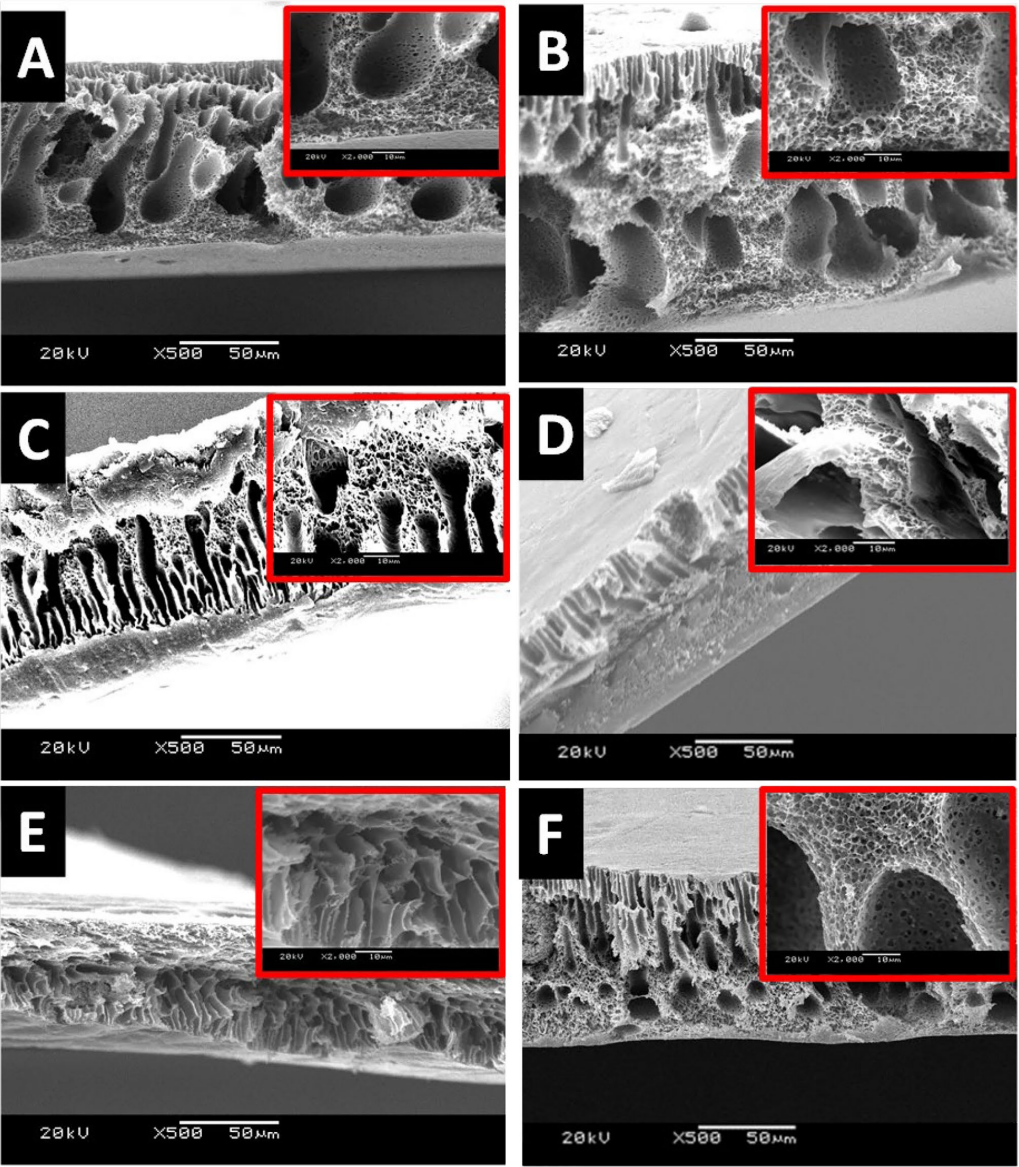
SEM cross-section images of the blank PES and the magnetic mixed matrix PES membranes that were prepared using 1 NMP: 9 DMF solvents mix without (**A,C,E**) and with (**B,D,F**) 0.1 wt% of Lithium chloride. The blank PES membrane (**A,B**), the magnetic mixed matrix PES membranes using Fe_10_Ni_90_ (**C,D**), and Fe_20_Ni_80_ (**E,F**) alloys were imaged at × 500 and × 2000 magnifications.

The cross-section images shown in Fig. 5 illustrate the formation of an asymmetric structure composed of a skin layer over a more open porous structure (i.e., selective and supporting layers). The change in the color of the top and the bottom of the mixed matrix PES membranes proposed that most of the alloys segregate into the skin layer. This is also supported by not noticing alloys existence within the cross-section images. Also, by the naked eyes, it was noticed that the mixed matrix PES membranes’ surfaces became jaggier whereas their bottom is smooth. As noticed, the cross-section illustrated thee porous shapes; the long porous next to the dense skin layer. The tiny pores network structure is rounding bigger pores/holes. This unique structure was noticeable in all the fabricated magnetic mixed matrix membranes.

**Figure 6:**
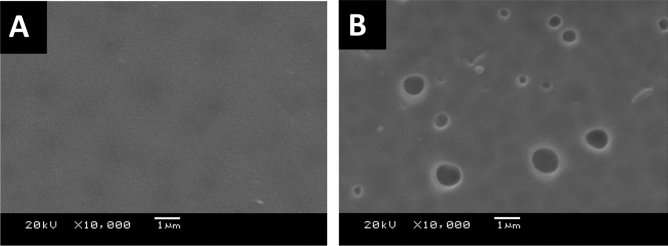
SEM images of the front (**A**) and the back (**B**) of the blank PES membranes that were prepared using 1 NMP: 9 DMF solvents mix with Lithium chloride.

Although using Lithium chloride as pore former, the dense nonporous skin layer (front side) was formed as shown in Fig. 6A. but the pores were shown in the SEM of the backbone (support back side) of the membrane as shown in Fig. 6B. Also, there is no pores were shown on the surface of the fabricated mixed matrix membranes as shown in SEM (Fig. 4).

### Membranes’ thickness and roughness

**Figure 7:**
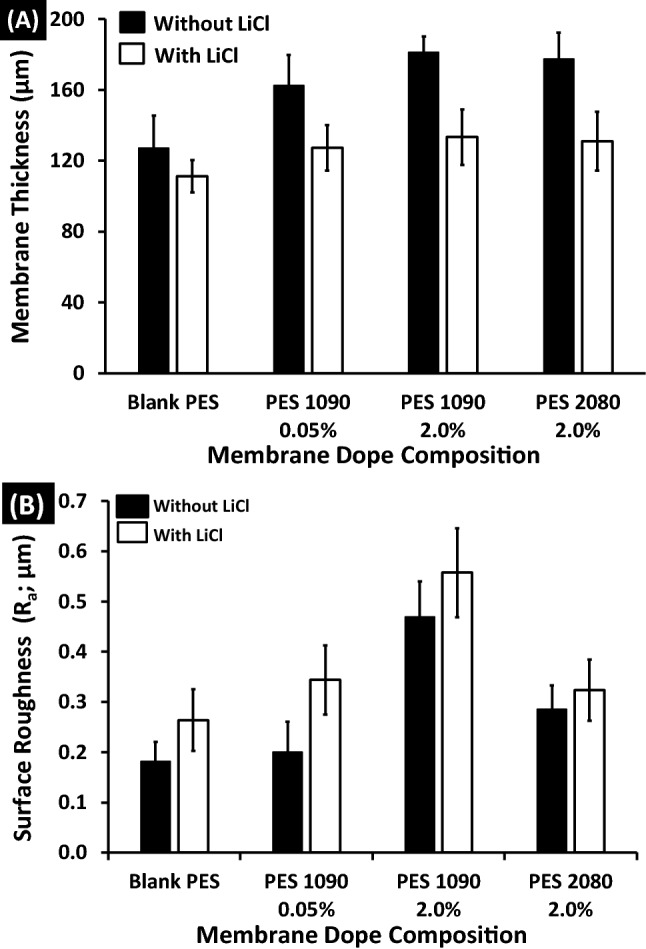
Surface thickness (**A**) and membrane roughness (**B**) of both the blank PES and the magnetic mixed matrix PES membranes as a function of membrane dope composition; wt% alloy concentration, in case of absence (black bars) and in case of using 0.1 wt% of Lithium chloride (white bars) in the membrane dope.

The as-cast thickness was 350 µm. After coagulation of the membrane dope, the thickness lost around 41–58% and 65–63% of their as-cast thickness in case of absence and presence of Lithium chloride, respectively as shown in Fig. 7A. The addition of iron–nickel alloys affects slightly the membrane thickness. As noticed, there are no significant differences in the measured membrane thickness as a function of the different alloys’ morphology and/or concentrations of the used condition in this work.

The surface roughness of the membranes has an important effect on the membrane characteristics; it is linked to the contact angle of the membrane and its hydrophilicity^42^ in which the surface roughening tends to increase the measure of the static water contact angle. The arithmetic average of the roughness profile (Ra) is calculated as the average roughness of surfaces measured by microscopic peaks and valleys. As shown in Fig. 7B, the absence of Lithium chloride highlighted the effect of adding iron–nickel alloys on the measured membrane roughness that increased significantly with a high concentration (2%) of Fe_10_Ni_90_ alloy (the surface roughness of the mixed matrix PES membrane is higher than the surface roughness of the blank PES membrane by 161%) rather than Fe_20_Ni_80_ alloy (surface roughness of the mixed matrix PES membrane is higher than the surface roughness of the blank PES membrane by 61%). This can be related to the different morphologies of the used two alloys^40^. In general, using Lithium chloride and its effect on creating voids affect the membrane surface to be slightly rougher than the same membrane that prepared without using Lithium chloride in the membrane dope.

### Membrane hydrophobicity

**Figure 8:**
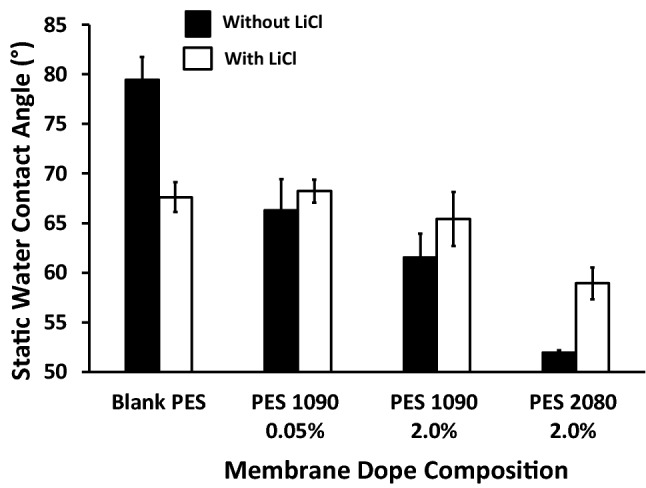
The static water contact angle of the blank PES and the different magnetic mixed matrix PES membranes composition in case of without (black bars) and with (white bars) using 0.1 wt% of Lithium chloride in the membrane dope.

The static water contact angle on both the blank PES and magnetic mixed PES membranes was measured and presented in Fig. 8. The addition of 0.1 wt% Lithium chloride into the blank PES resulted in about a 15% reduction in the measured static contact angle. In the absence of Lithium chloride, the addition of magnetic iron–nickel alloy resulted in a significant reduction in the measured static water contact angle as a function of the used Fe_10_Ni_90_ alloy concentration (16.5 and 22.5% for 0.05 and 0.2 wt%, respectively). Moreover, the different alloy morphology has a different effect at the same used concentration. The 2 wt% Fe_20_Ni_80_ alloy has resulted in a reduction of up to 34.6% compared to a 22.5% reduction in the case of adding the same concentration of Fe_10_Ni_90_ alloy. In the case of using Lithium chloride, the effect of Lithium chloride was diminished or even inverted by the effect of adding the alloys in which the measured static water contact angle of different mixed matrix PES membranes is slightly higher than the measured static water contact angle of the same mixed matrix PES membranes but without using the Lithium chloride (up to 13% difference) in the membrane dope.

### Membrane porosity

According to the Laplace equation, the penetrating pressure is linear to the solvent surface tension. So, a solvent with low surface tension is the better choice of the used solvent to determine the membrane porosity. The surface tension of distilled water and ethanol is 72.8 and 21.6 mN/m, respectively. Also, in general, any solvent that effectively wets the membrane can be used, but a smaller molecule (ethanol vs. distilled water) will penetrate more pores and the result will be somehow higher by using ethanol than by using distilled water, especially if the membrane has very small pores (smaller than mesoporous). For that, measuring porosity using distilled water can be related to large-size pores, whereas porosity using ethanol can be related to all ranges of pores.

**Figure 9:**
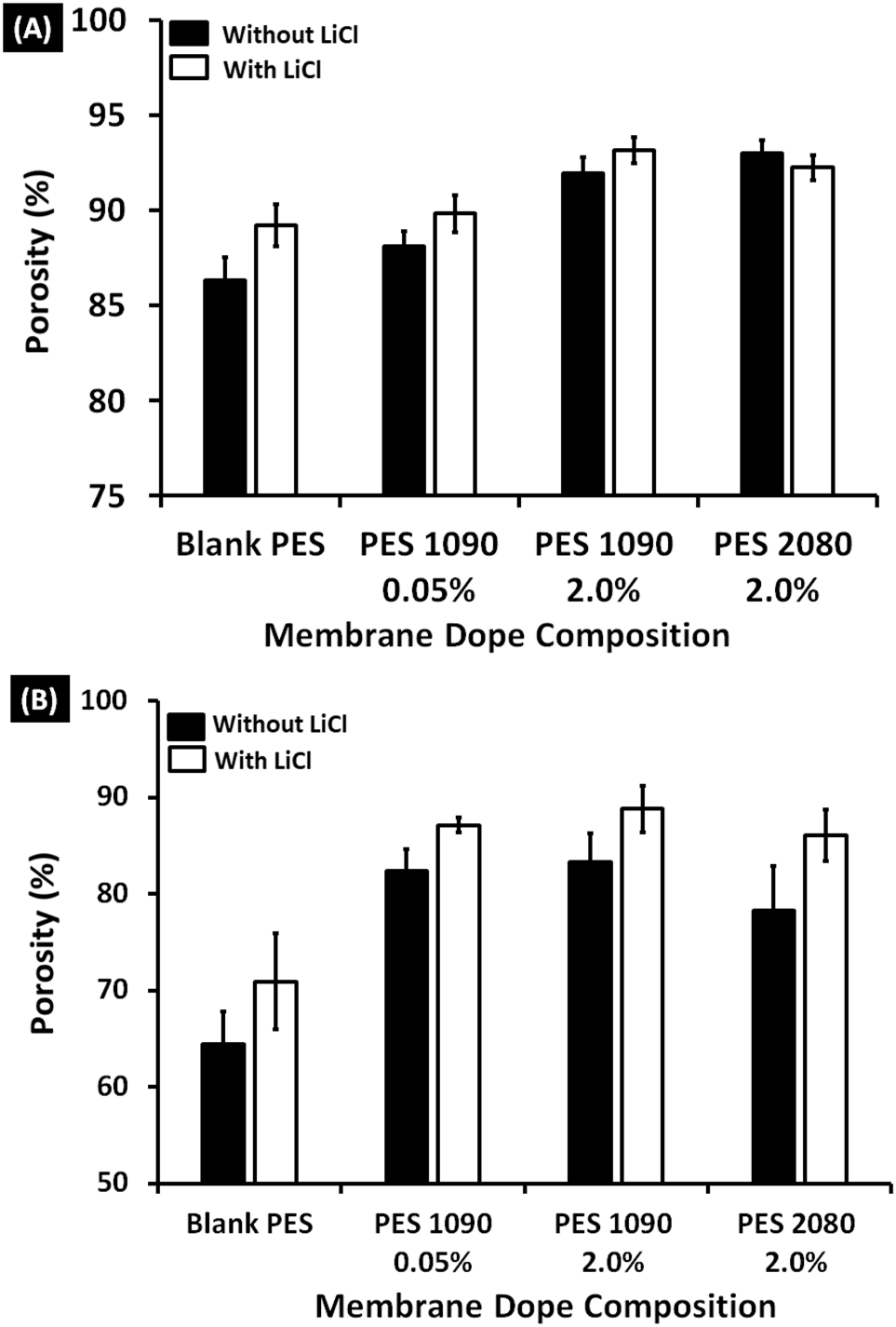
Porosity (%) of the blank PES and the magnetic mixed matrix PES membranes in absence (black bars) and precence (white bars) of 0.1 wt% of Lithium chloride in the membrane dope using absolute ethanol (**A**) and distilled water (**B**).

As shown in Fig. 9, the porosity of the blank PES and the magnetic mixed matrix PES membranes were measured using ethanol and distilled water with ranges of 86–93% and 64–88%, respectively. Adding the magnetic alloys affects positively the membrane porosity. But, the effect of the Lithium chloride and the different morphologies on the overall porosity is very slight. The effect of using Lithium chloride is noticeable on the large-size porosity that measured by distilled water.

### Membrane characterization (bulk properties)

#### X-ray diffraction (XRD)

**Figure 10:**
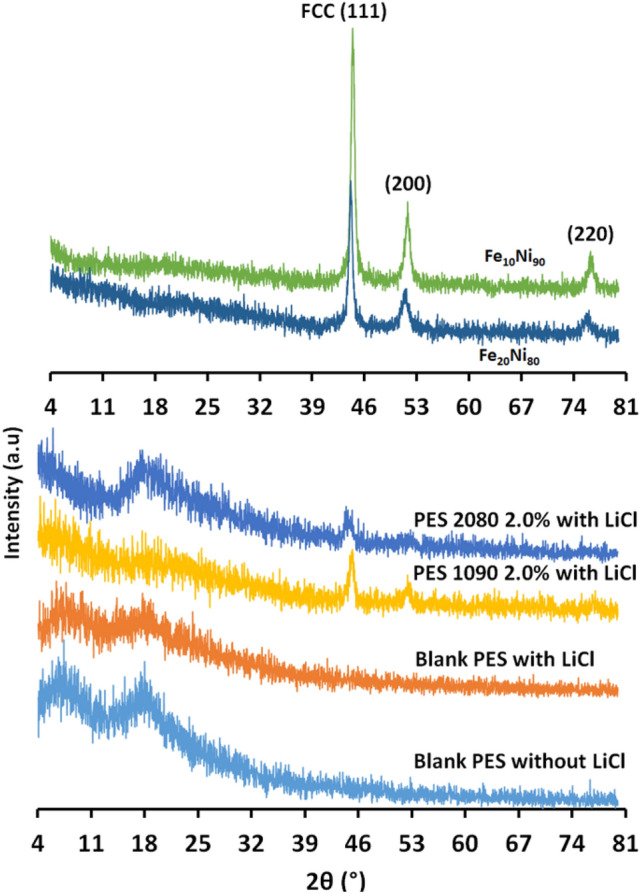
XRD analysis of the used magnetic iron–nickel alloys as filler, the blank PES without and with adding 0.1 wt% of Lithium chloride in the membrane dope, and the magnetic mixed matrix PES membranes in case of using 0.1 wt% of Lithium chloride in the membrane dope; PES 1090 2.0% W and PES 2080 2.0% W.

X-ray diffraction (XRD) in Fig. 10 showed the presence of the broad peak around 18° that characterizes the PES polymer as well as the peaks located at 44.41°, 51.71°, and 76.21° that can be indexed to (111), (200), and (220) planes of the crystalline face-centered cubic (fcc) FeNi_3_ alloys^40^, indicating the successful impeding the iron–nickel alloys inside the PES matrix. The obtained result supports the successful formation of mixed matrix PES membranes.

### Thermogravimetric analysis (TGA)

**Figure 11:**
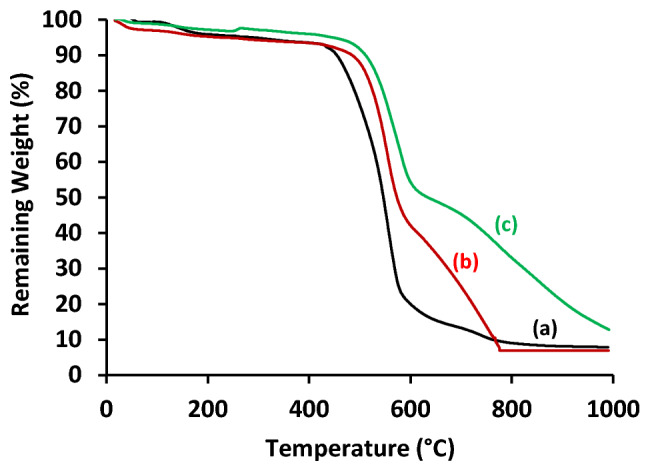
TGA analysis of the blank PES membrane (a) and the magnetic mixed matrix PES membranes (b; PES 1090 2.0% and c; PES 2080 2.0%) in case of using 0.1 wt% of Lithium chloride additive.

Figure 11 shows the TGA analyses for the blank PES and the magnetic mixed matrix PES membranes. The initial weight loss below 200 °C corresponded to the removal of moisture and/or the used solvent in the membrane fabrication; this was less than 4%. The weight loss of the blank PES membrane is around 450 °C (93 wt% remaining) which can be attributed to sulfur dioxide cleavage and ether bond cleavage. At higher temperatures; the second thermal degradation stage is starting around 575 °C (26 wt% remaining) and the backbone (benzene ring) decomposes. This temperature was slightly increased to around 588 °C and 606 °C with 44.7 and 53 wt% remaining for PES 1090 2.0% and PES 2080 2.0% magnetic mixed matrix PES membranes, respectively. The change in the slope of the degradation of the magnetic mixed matrix PES membranes than the blank PES membrane can be an indication of thermal stability of the magnetic mixed matrix PES membranes. However, it seems PES 2080 2.0% magnetic mixed matrix membrane (line c) is more stable than the PES 1090 2.0% magnetic mixed matrix membrane (line b) at temperature higher than 600 °C.

### The elemental composition

**Figure 12:**
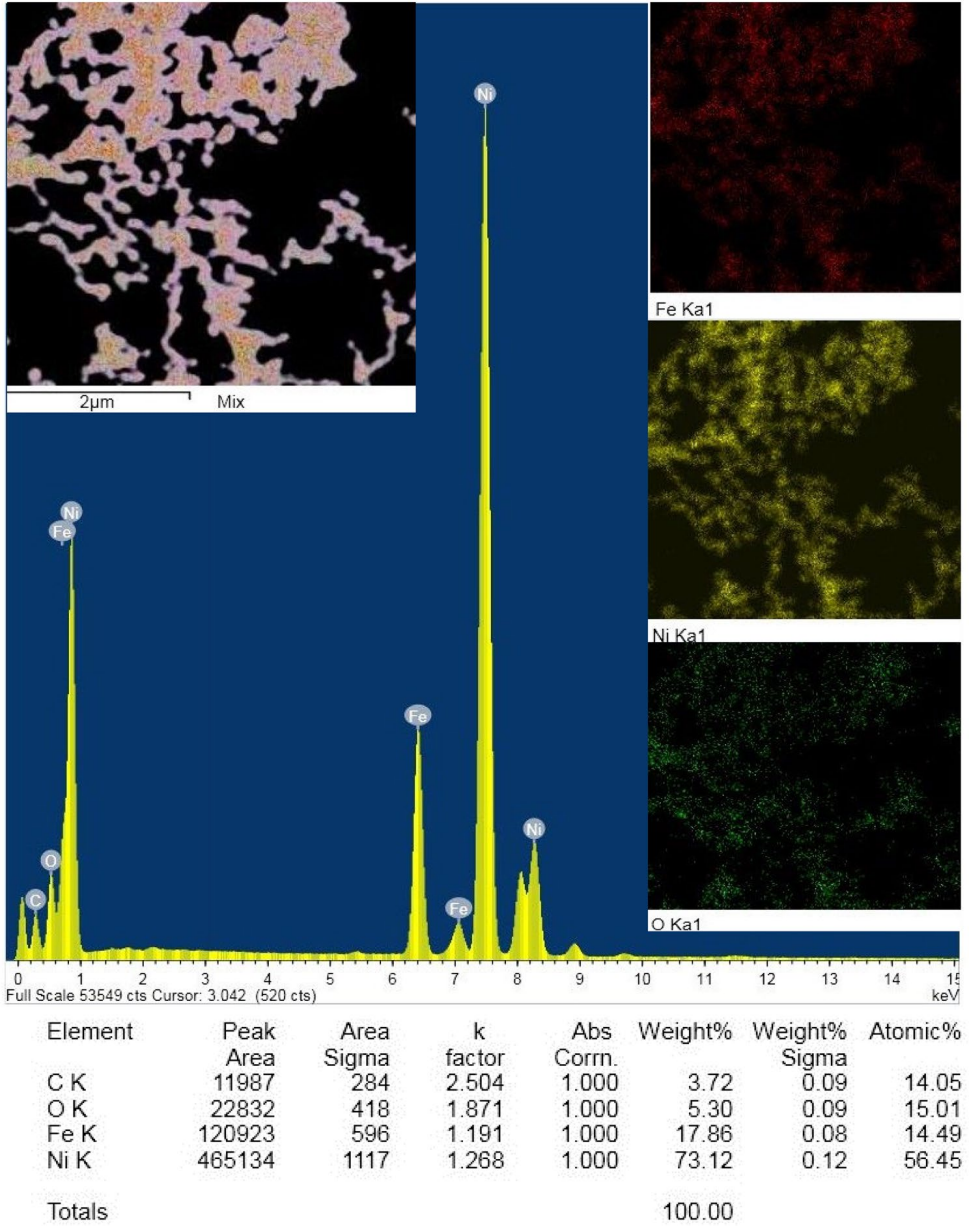
TEM elemental mapping of PES 2080 2.0% W magnetic mixed matrix membrane.

The elemental composition of blank PES and the magnetic mixed matrix PES membranes in cases of absence and using Lithium chloride in the membrane dope were investigated by EDX analysis equipped in SEM (Table 2) and TEM (Table 3) where both the at% and mass% are shown. In SEM, the source of X-rays is a sphere with a diameter of about 1000 nm, whereas, in TEM, the source of X-rays is a disk with a beam's diameter around 50–100 nm section. It is well known that EDX is not a tool for precision chemical analysis, it is just an instrument for estimation of elements distribution in a specimen. It was observed that the blank PES membrane contained oxygen, sulfur, carbon, and other traces of chloride, which can be attributed to the presence a trace element form the used Lithium chloride (The detector of the used EDX did not detect Lithium element). The magnetic mixed matrix PES membranes contained oxygen, sulfur, carbon, iron, nickel, and trace of chloride. The decrease of sulfur content in the magnetic mixed matrix PES membranes may be a sign of the formation a new composite of PES and the iron–nickel alloys. EDX equipped with the TEM illustrates more clear indication of including the iron–nickel alloys inside the magnetic mixed matrix PES membrane due to much lower beam thickness, the used molar ratios 1:9 and 2:8 is in full agreement with the determined iron and nickel contents in PES 1090 2.0% W (i.e.; 8.88/75.49 At% and 9.61/ 85.91 wt%) and PES 2080 2.0% W (i.e.; 14.4/54.65 At.% and 16.67/66.52 wt%) membranes, respectively. Also, the elemental analysis mapping shown in Fig. 12 supports the uniform distribution of the used iron–nickel alloy inside the magnetic matrix PES membrane.

**Table 2:** EDX analysis of the fabricated membranes using SEM equipment.

Membrane code	SEM EDX analysis											
	Atomic%						Mass%					
	Fe	Ni	O	S	C	Cl	Fe	Ni	O	S	C	Cl
Blank PES WO	0.0	0.0	6.96	11.45	81.59	0.0	0.0	0.0	7.63	25.18	67.19	0
Blank PES W	0.0	0.0	21.88	11.10	66.60	0.42	0.0	0.0	22.26	22.63	54.16	0.95
PES 1090 2.0% WO	0.24	0.14	18.66	9.97	70.99	0.0	0.57	0.55	20.07	21.49	57.32	0.0
PES 1090 2.0% W	0.98	4.84	14.31	4.10	75.76	0.01	3.41	17.66	14.23	8.17	56.53	0.0
PES 2080 0.02% WO	1.32	0.96	10.68	11.73	75.32	0.0	4.65	3.55	10.81	23.79	57.21	0.0
PES 2080 2.0% W	0.13	1.51	21.47	7.94	68.78	0.17	0.46	5.83	22.50	16.69	54.12	0.40

**Table 3:** EDX analysis of the fabricated membranes using TEM equipment.

Membrane code	TEM EDX analysis											
	Atomic%						Mass%					
	Fe	Ni	O	S	C	Cl	Fe	Ni	O	S	C	Cl
Blank PES W	0.0	0.0	8.16	0.05	91.41	0.38	0.0	0.0	10.54	0.13	88.52	0.81
PES 1090 2.0% W	8.88	75.45	16.92	1.30	0.0	0.06	9.61	85.91	5.25	0.81	0.0	0.04
PES 2080 2.0% W	14.4	54.65	11.9	16.13	0.0	2.92	16.67	66.52	3.95	10.72	0.0	2.14

### Mechanical strength

**Figure 13:**
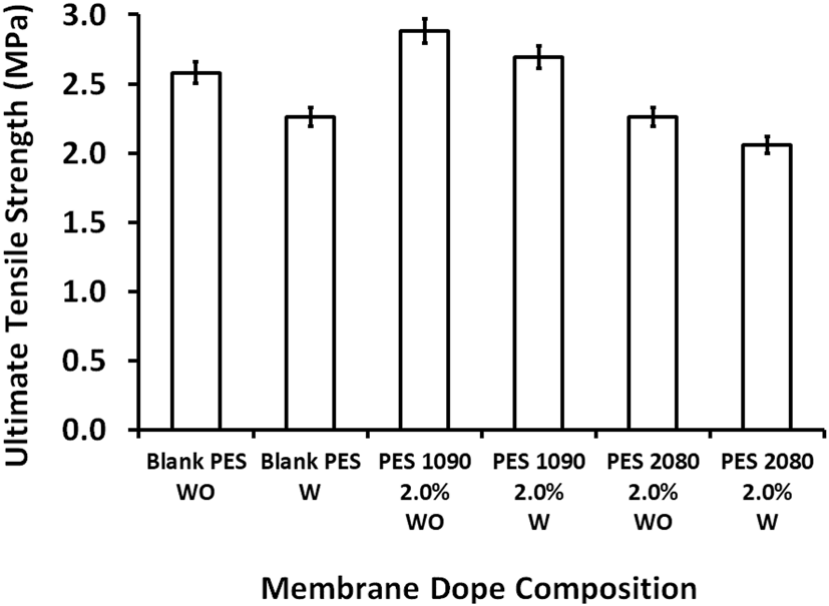
Ultimate tensile strength as a function of the membrane dope composition of both the blank PES and the magnetic mixed matrix PES membranes without (WO) and with (W) 0.1 wt% of Lithium chloride additive in the membrane dope.

As shown in Fig. 13, the ultimate tensile strength of the blank PES membrane has slightly decreased with using the Lithium chloride in the membrane dope, however, the addition of magnetic iron–nickel alloys with different microstructures has differently affected the mechanical strength of the fabricated magnetic mixed matrix PES membranes. The starfish-like; Fe_10_Ni_90_, affects positively the membrane strength whereas the necklace-like; Fi_20_Ni_80_, affects it negatively. This can be attributed to the good hanging of the cones of the starfish-like iron–nickel alloy with the polymeric matrix rather than the crowded necklace-like iron–nickel alloy as indicated by TEM and SEM imaging.

### Vibrating sample magnetometer (VSM) analysis

**Figure 14:**
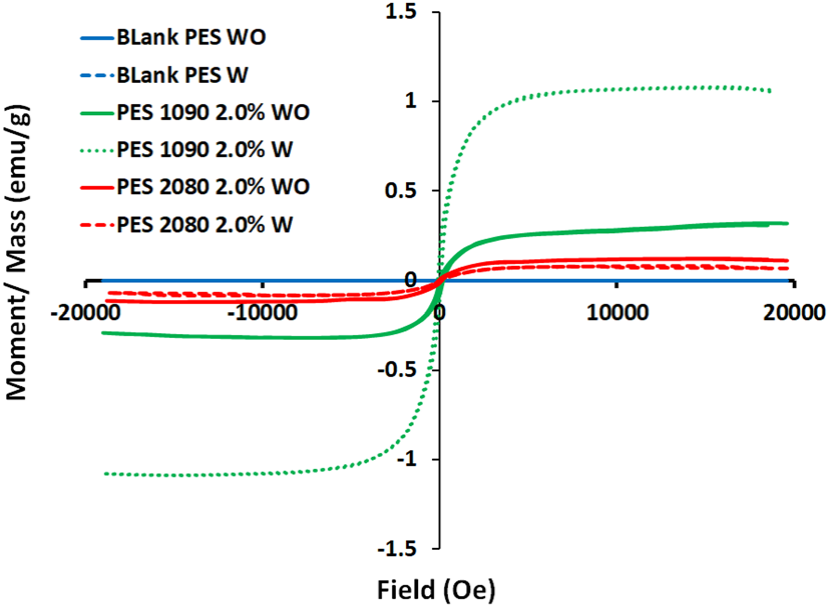
The M-H hysteresis loops of the bank PES and the magnetic mixed matrix PES membranes (PES 1090 2.0% and PES 2080 2.0% without and with using 0.1 wt% of Lithium chloride additive in the membranes dopes.

The M-H hysteresis loops of the blank PES and the magnetic mixed matrix PES membranes are shown in Fig. 14. The magnetic mixed matrix PES membranes form an S-shape to show their magnetic properties, but the blank PES membranes do not form a clear S-shape. The effect of Lithium chloride on the membrane magnetic properties is unique and not completely understood^43^.

**Figure 15:**
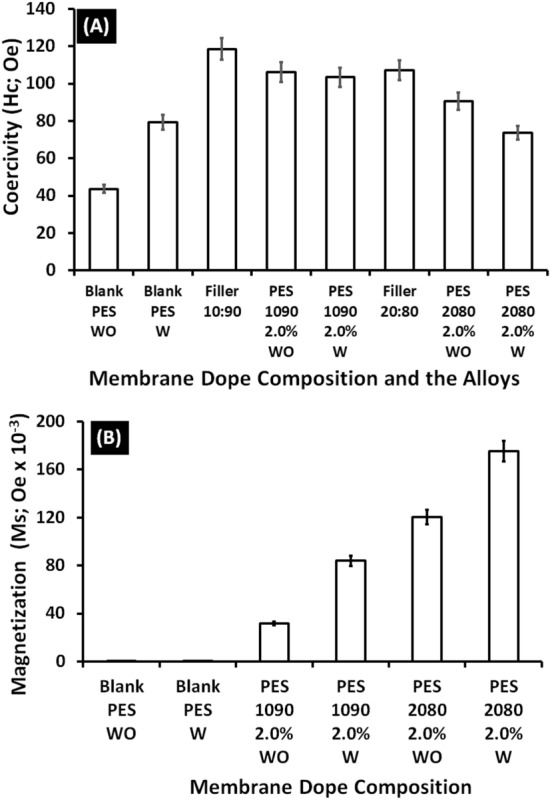
(**A**) Coerceivity (Hc; Oe) and (**B**) magnetization (Ms) of the used alloys (Fe_10_Ni_90_ and Fe_20_Ni_80_), the bank PES and the magnetic mixed matrix PES membranes (PES 1090 2.0% and PES 2080 2.0%) without and with using 0.1 wt% of Lithium chloride additive in the membrane dope.

The coercivity (Hc) and the magnetization (Ms) of the used alloys (Fe_10_Ni_90_ and Fe_20_Ni_80_), the bank PES, and the magnetic mixed matrix PES membranes without and with using Lithium chloride in the membranes dopes are shown in Fig. 15. The results of the magnetic vibration of the fabricated magnetic mixed matrix PES membranes showed high coercivity (Hc; Oe) than the blank PES membrane (about 147% improvement) in case of the absence of Lithium chloride additive. The blank PES in the case of using Lithium chloride in the membrane dope showed an improvement in the coercivity of the membrane by a ratio of 83% which can be an indication of the non-complete removal of the Lithium chloride additive during the demixing process (membrane solidification process). The presence of some Lithium chloride in the membrane was also confirmed by EDX analysis. For that, in the case of using Lithium chloride as an additive, the improvement in the coercivity of the magnetic mixed matrix PES membrane was only 30% of the blank PES membrane.

On the other hand, the magnetization of the magnetic mixed matrix PES membrane in the case of using Lithium chloride additive is almost three times the magnetization of the same magnetic mixed matrix PES membrane without using Lithium chloride additive in the membrane dope. The blank PES membrane without and with using Lithium chloride additive in the membrane dope showed almost no magnetization properties. According to the different iron–nickel alloys, the magnetic mixed matrix PES membranes that contained Fe_20_Ni_80_ showed higher magnetization and lower coercivity than the magnetic mixed matrix PES membranes contained Fe_10_Ni_90_ alloy. Regarding the required application, the gas separation without applying an external magnetic field on the separation cell highlights the coercivity property for better performance of the fabricated magnetic mixed matrix PES membranes.

### Oxygen transmission rate (OTR)

**Figure 16:**
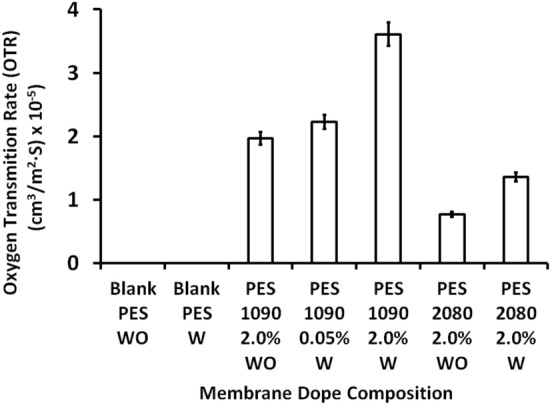
Oxygen Transmission Rate (OTR) of the bank PES and the magnetic mixed matrix PES membranes without and with using 0.1 wt% of Lithium chloride additive in the membrane dope.

Permeability decides the quantity of the penetrant molecules which have passed through the membrane. The permeate flux rate is the rate of transport of gas molecules in a given thickness of the material. Figure 16 shows the oxygen transmission rate which is the measurement of the amount of oxygen gas that passes through a barrier over a given period. The blank PES membrane without and with using Lithium chloride in the membrane dope does not give results for oxygen permeation. Adding the magnetic alloys resulted in oxygen transmission. The magnetic mixed matrix PES membranes containing starfish-like Fe_10_Ni_90_ alloy that shows higher efficiency in oxygen transmitting than the magnetic mixed matrix PES membranes that containing necklace-like Fi_20_Ni_80_ alloy. Moreover, the oxygen transmission rate is a function of the alloy concentration for both the two alloys. On the other hand, it seems that Lithium chloride additive enhances the transmission of oxygen due to increasing the porosity in the supporting porous layer.

## General discussion

Selectivity is a key parameter for achieving high product purity at high recoveries^44^. The separation mechanism of porous (inorganic) membranes is based on either molecular sieve (i.e., the smaller molecules are permeated through the membrane pores, while the larger ones are blocked) or adsorption-selective that depends on their adsorption properties and interaction with the membrane material^45–47^.

The desired membrane morphology in the gas separation is a spongy structure with considerably low membrane thickness^48^. Besides, the selectivity of a membrane is depending on the type of polymeric material used in the membrane fabrication.

In polymeric membranes, the transport of gas molecules takes place due to the random molecular motion of individual gas molecules. Mostly polymeric membrane show good selectivity due to the presence of low free volume companies with low permeability as shown in the trade-off of Robeson upper bounds^49^.

Glassy polymers are used as materials for gas separation due to their higher selectivity in contrast to the rubbery polymers which have higher permeability, but low selectivity. Polyethersulfone (PES) is a highly highlighted glassy polymer material in gas separation membranes because it has a diphenylene sulfone repeating unit as shown in Fig. 1 that forms a thermoplastic polymer with a rigid backbone that has a high degree of immobility, high mechanical, thermal, and chemical strength, good creep resistance, and high dimensional stability^35^. However, the tradeoff limitations and plasticization of the polymer chains are still challenges that need to be adapted^37^. For that, PES has been chosen for improving its application in gas separation in this work.

**Figure 17:**
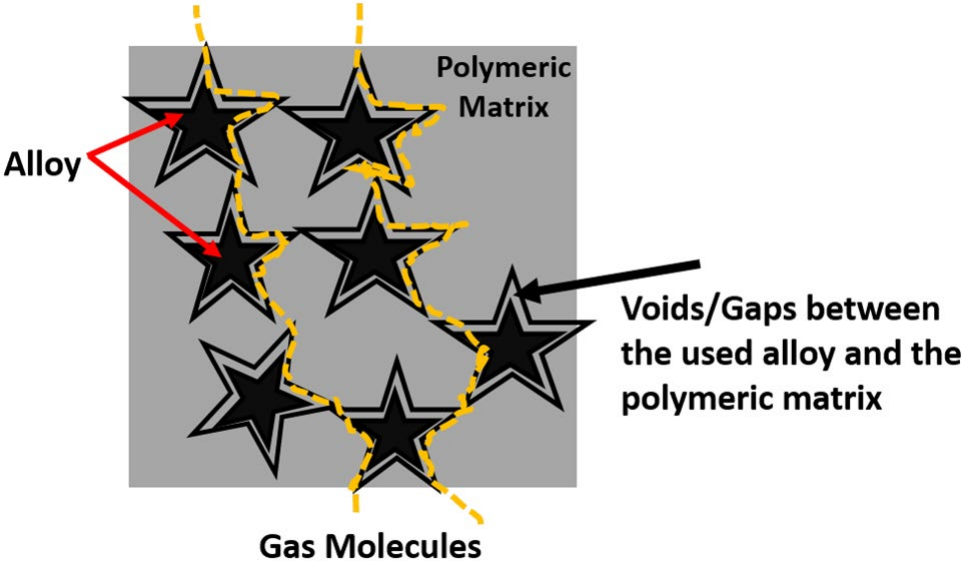
Schematic Diagram illustrate the role of the gabs surrounding the alloy.

Flat sheet blank PES and magnetic mixed matrix PES polymeric membranes were prepared in this work by solution casting and phase inversion method and the prepared magnetic iron–nickel alloys were embedded as fillers. All the fabricated membranes were characterized using different analyses techniques that highlighted the following points:

1. The used casting knife attracted the alloys near/into the surface and the magnetic fillers were pulled up to the membrane surface in the direction of the knife move. This keeps the magnetic filler concentrated underneath the membrane surface and minimizes filler sedimentation. This attraction between the used knife and the filler canceled the need to apply an external magnetic field during membrane casting.
2. Although Lithium chloride was used to work as pore former, it can also generate a denser surface structure due to the formation of complexes with NMP solvent, which can significantly increase the dope solution’s viscosity as described by other researchers^43^. This can play as a kinetic hindrance during the phase inversion process that leads to the surface layer becoming denser. The dense nonporous skin layer was noticed in SEM (Fig. 4); more pores were illustrated in the membrane sponge support layer in the cross-section (Fig. 5). Whereas, the created pores by the used Lithium chloride were shown in Fig. 6 at the back of the membrane.
3. Porosity is the amount of total void space present in the membrane. A higher number of pores will reduce the selectivity and a lesser number of pores will improve the selectivity and decrease the permeability. The porosity also contributes to the mechanical strength of the membrane, flux rate, and solubility of gas molecules. In this work, the oxygen transmission rate is influenced by the affinity of the oxygen toward the magnetic fillers that increase the transition rate of the oxygen with an increase in the concentration of the Fe_10_Ni_90_ alloy. However, this affinity is different with different filler composition and consequently their morphologies and magnetic properties; PES 1090 2% W showed OTR 2.6-fold higher than PES 2080 2% W; although both incorporated magnetic mixed matrix membranes showed no significant difference in their determined porosity.
4. The poor compatibility of the filler surface and the polymer has been proposed^50^ as a reason for the increase in membrane permeability while maintaining the selectivity within the original range. The gas diffusion path was shortened and thus, the apparent gas diffusivity and permeability have increased^50,51^; In full agreement with this proposed effect and the observation and the obtained data in this work, the hydrophilic property of the fabricated alloys and the relatively hydrophobic PES matrix proposed low compatibility between their surfaces with the creation of gaps (i.e., The polymer chains could not tightly contact the filler surface, thus forming a narrow gap surrounding the filler) as illustrated in Fig. 17. This effect was assisted by the effect of Lithium chloride pore former. Hence, the adhesion between the organic matrix and the inorganic filler particles should be studied in-depth.
5. The thickness of the membrane reduces the permeability of gas molecules in which membrane with large thickness will give greater distance for the gas molecules to travel thus results in reducing the gas diffusion and solubility through the membrane. Membrane thickness (around 120 µm) that was used in this work may contribute in the enhanced the gas permeation in the fabricated mixed matrix PES membranes.
6. In full agreement with previous studies^51^, both of the coercivity and the saturation magnetization depended on the composition and microstructure of the magnetic alloys. The values of saturation magnetization of the fabricated magnetic mixed matrix PES membrane were lower than that of bulk magnetic alloys. This is can be attributed to the influence of polymeric chains on the magnetic alloys’ properties.

## Conclusions

Although researchers have succeeded in preparing many magnetic mixed matrix membranes from different polymers and inorganic magnetic fillers, there are several drawbacks that may affect their membrane’s performance and this work presented a solution for these drawbacks that include (1) The used rare magnetic filler such as praseodymium or neodymium is a very expensive magnetic filler which impedes its application on a large scale, (2) The used iron-oxide nanoparticles as filler in magnetic mixed matrix membranes need to apply an external magnetic field during the fabrication process to minimize sedimentation of the filler in the membrane’s back and during the separating process because the iron-oxide nanoparticles lost their magnetization once the magnetic field moved away (i.e., the membrane lost its affinity toward the targeted molecules), (3) Most methods of preparation do not provide well-dispersed magnetic fillers in the formed membranes.

In this work, the presented novel magnetic mixed matrix PES membranes have high coercivity up to 106 (emu/g) with 3.61 × 10^–5^ cm^3^/cm^2^·s OTR compared to non-oxygen permeable blank PES membranes. They combine the advantages of both low-cost common polymers and low-cost simple-prepared inorganic fillers and enable the use of it in a wider range, and are more efficient in different applications without applying an external magnetic field during either the membrane casting or the separation process. The presented magnetic mixed matrix PES membranes open new areas for the use of mixed matrix membranes in different applications and on an industrial scale.

## Data Availability

All data generated or analyzed during this study are included in this published article.
